# Integrated Nonpharmacological Intervention for Patients with MCI—A Preliminary Study in Shanghai, China

**DOI:** 10.5334/ijic.5706

**Published:** 2022-03-04

**Authors:** Hui Yang, Yinghong Liu, Honglin Chen, You Yin

**Affiliations:** 1 Department of Sociology, Minzu University of China, Beijing, China; 2 Department of Social Work, Fudan University, Shanghai, China; 3 Department of Social Sciences, University of Eastern Finland, Finland; 4 Department of Social Work, Fudan University, China; 5 The Department of Neurology, Changzheng Hospital, the PLA Naval Medical University, China

**Keywords:** mild cognitive impairment, social work, integrated nonpharmacological intervention, multicomponent nonpharmacological intervention approach

## Abstract

**Objective:**

Mild cognitive impairment is a transitional state between normal aging and Alzheimer’s and is an internationally recognized pre-stage state of Alzheimer’s. It is the starting point for early detection and intervention in dementia. An empirical study was conducted to determine how medical social workers provide intervention services to older adults with mild cognitive impairment, specific strategies for change, and the effectiveness of the intervention.

**Methods:**

This was a quasi-experimental intervention study.

**Conclusion:**

Through integrated intervention, the development of cognitive impairment can be significantly delayed, cognitive abilities can be improved (*p* < .05), and decline in daily living abilities can be significantly delayed (*p* < .05). At the same time, the mental health of participants can be improved to a certain extent.

## Introduction

With the accelerated aging of the world’s population, Alzheimer’s disease is becoming a global public health problem. Regarding this major problem in the medical field, there has been neither the emergence of specific drugs nor universally effective treatment options for Alzheimer’s disease; no cases have been cured by modern medical methods. Therefore, domestic and foreign scholars have focused on risk factors and areas of early detection, diagnosis, and treatment for Alzheimer’s [1]. Through ongoing research, scholars have discovered that in the normal aging process, there is a stage known as mild cognitive impairment (MCI). This state is defined by researchers as a precursor to Alzheimer’s among older adults. These older people with MCI have cognitive dysfunction that is more severe than the normal decline in their age range but not enough to meet the criteria for a medical diagnosis of dementia. The duration of this stage is not fixed; the disease’s development is affected by many factors, and it has a high conversion rate to dementia. According to some studies, the average annual conversion rate from MCI to dementia is 10% to 15%, whereas the conversion rate in 5 years is as high as 50%, which is far higher than among normal older people [2]. According to statistics from the National Health Commission, with each additional 5 years of age among older people in China, the risk of dementia increases by 1.85 times [3]. Though the fundamental problem faced by older people with cognitive impairment is cognitive functional decline, emotional and daily life problems also cause physical and psychological challenges. Meanwhile, the loss of the ability to perform activities of daily living (ADL) in the later stage of the disease often necessitates intensive companionship and care. This can place a heavy burden on family members and society simultaneously; therefore, it highlights the importance of early diagnosis and intervention for people with MCI. Nonpharmacological intervention to delay the development of the disease has become an important research topic in such fields as social work for older people and medical social work. In the field of social work with older people, social workers use relevant theories to intervene to different degrees and in different types of cognitive dysfunction through case work, group work, and community work. But there is still a lot of room for development.

The current domestic research on MCI is insufficient, and most research has been in the medical and nursing fields. Older adults who might benefit most from nonpharmaceutical intervention are concentrated in communities, nursing homes, or inpatient groups. In those studies, the researchers either employed nonpharmacological intervention methods or pharmacological intervention only. Very few studies tried to combine the two approaches in one project. This study aimed to verify the effectiveness of an integrated nonpharmacological social work group intervention together with medical intervention in delaying the development of cognitive impairment and to explore a hospital–family dual model led by medical social workers. The project also involved a follow-up process for patients to continuously strengthen the intervention in their family.

## Study on Intervention of MCI

Currently, the treatment for Alzheimer’s and MCI can be divided into medicinal therapy methods and nonpharmacological interventions. Research indicates that drugs have a positive effect on slowing down the incidence of MCI and conversion to Alzheimer’s. At the same time, nonpharmacological interventions such as psychological interventions and cognitive training also have a positive influence on patients with MCI, which can improve their quality of life by slowing down the rate of disease development.

### Drug treatment

Drug treatment includes drugs that target symptoms of memory decline, brain atrophy, and reduced mobility like ChEI, donepezil, reminyl, and rivastigmine; drugs that target risk factors like statins, hyperhomocysteinemia drugs, antidiabetes medicine, and antihypertensive drugs; and drugs for improving nutrition like medium-chain triglycerides, antioxidants, and ω–3 aliphatic acid. Other therapy methods use traditional Chinese medicine for MCI, including Chinese herbs such as polygala tenuifolia, gingko, epimedium herb, acorus gramineus soland, ginseng, and so on, along with traditional Chinese medicinal compounds such as fructus alpiniae oxyphyllae and tranquillizer, expectorant mixture activating collaterals, tonifying qi, warming yang and compounds that promote blood circulation [4]. However, clinical studies have shown that the effect of drug treatment on alleviating disease development is not obvious, and some drugs have obvious side effects, which can negatively affect older people [5].

### Nonpharmacological intervention

Currently, nonpharmacological intervention is considered one therapy method that has no side effects and can be accepted by older people, not only decreasing negative symptoms but also slowing the process of development from MCI to Alzheimer’s [6]. The nonpharmacological intervention therapy method focuses on not only the recovery of cognitive ability, but also the psychosocial health of the patient. The most utilized methods include individual recognized training, exercise therapy, music therapy, and psychological interventions.

### Social work intervention in MCI

Regarding studies on social work with older adults with cognitive impairment, the field is dominated by research on severe Alzheimer’s [7]. Prior research mainly involved exploration of social work interventions seeking to prevent dementia [4], social work intervention paths for MCI [8], late intervention and hospice care for cognitive disorders [9], etc. Participants in domestic research are mainly older adults living in the community or nursing institutions [10]. Also, the total sample size of the intervention group in these studies has been fairly low, which may be related to the limitation of the group work model used in research regarding the number of participants for the best treatment effect.

Social work interventions for MCI have become a hot spot for nonpharmacological interventions for MCI in recent years. In the study of social work interventions among older adults with MCI, theories related to social work have also been applied and developed. There have been certain attempts—in case, group, and community work, for example—to analyze the needs of older adults in nursing homes and explore social work intervention approaches. These studies found that the quality of life of older adults can be improved by using social work case, group, and community work methods. One study found that social work treatment groups can not only bring therapeutic and rehabilitative effects for older adults with Alzheimer’s disease, but also reduce rejection and psychological harm through experience sharing and mutual assistance in group work [10]. In a decade-long study of many psychosocial interventions for patients with dementia, researchers found sufficient evidence that group-based cognitive stimulation can improve cognitive function, social interactions, and quality of life, highlighting the potential importance of group activities for improving the social integration of people with dementia [11].

In terms of specific intervention methods, one intervention strategy guided by a certain theory is more common; cognitive training therapy guided by Bandura’s learning theory [12] has been the most widely recognized and applied. The process of professional cognitive training is relatively boring and requires continuous strengthening of exercises, and the use of social work methods can make the intervention process more humane and easier to accept. The great difference between social work interventions and general nonpharmacological interventions is that social work interventions not only focus on improving the cognitive ability of patients, but also expect them to achieve psychological or spiritual improvement through the intervention, including gaining a sense of accomplishment, restoring self-confidence and value, relieving stress, regulating emotions, increasing social interaction, increasing utilization of social support, etc. Music therapy [10], remembrance (nostalgia) therapy [13], horticultural therapy [14], and play therapy [15] are often used in social work interventions and have been proven to have a good intervention effect [16]. Researchers who focus on social psychology should determine whether the intervention can enhance the function and happiness of the client. Most differences in the development of individual illness can be explained by the interaction between cognitive impairment and psychological and social factors, including the influence of personality, lifestyle and habits, stress coping styles, ability to regulate emotions, social support resources, and the ability of important others to provide emotional support and care. This view lays the foundation for the biopsychosocial model that has been widely accepted in related research, also known as the “rich model” or “human-centered” approach [16]. At the same time, it provides guidance for the development of psychosocial interventions for older adults with cognitive impairment.

In general, the exploration of domestic social work interventions in the field of cognitive impairment among older adults is still in the exploration stage, and there is a lack of effective interventions that are both professional and systematic. Compared with general nondrug treatment, how to highlight and identify the therapeutic factors of social work, especially group work, is also a problem that needs attention. In addition, the domestic social work field has performed insufficient research on the direction of cognitive impairment interventions, both in terms of the total amount of research and the sample size in specific studies; the significance and generalizability of service effects also need more empirical research support.

## Methods

**Figure 1:**
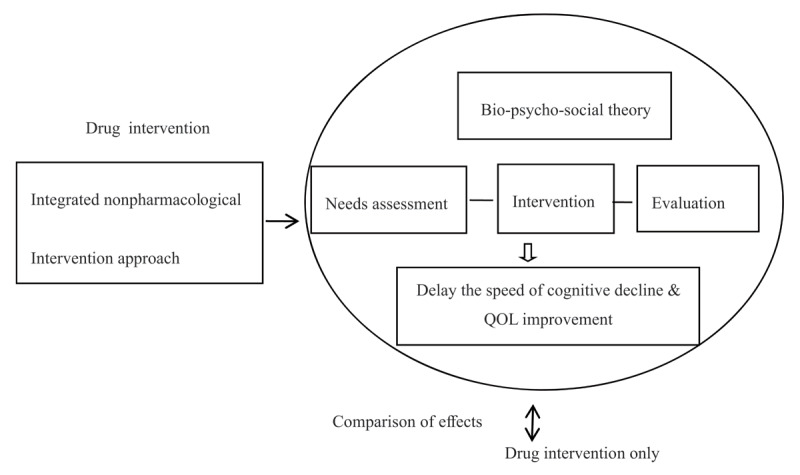
Research Framework.

In the data collection stage, we sought to discover research questions and assess the demands of patients. First, through the internship process in a neurology clinic, we extensively recruited patients with MCI and their families. Through observation and interviews, we developed a preliminary understanding of the typical characteristics, concerns, and potential needs of this group. Of note, most patients and family members do not understand the cause of the disease, the development process, etc., and there are feelings of helplessness and depression. At the same time, we analyzed general needs related to cognitive impairment, especially patients with MCI, through the literature, which mainly related to improving cognitive ability, delaying disease development, and improving psychological conditions. In addition, we conducted a systematic review of domestic and foreign research data on MCI to understand and summarize the current status of research on MCI for older adults at home and abroad; determine the effective nonpharmacological intervention methods and related social work intervention studies for the MCI population; determine the content, questions, and theoretical support of this research; and provide services based on the social work therapeutic group model. The research framework is shown in ***Figure 1*** above.

### Intervention study design

This intervention study used a quasi-experimental research design, with the goal of controlling irrelevant variables as much as possible. Two test groups and a control group were created, and the degree of cognitive impairment and psychosocial function of each group of patients with dementia was assessed at baseline with a professional scale. The test groups received conventional medical care combined with social work intervention, and the control group only received conventional medical care. After the intervention period, each group was tested again, and statistical analysis software was used to analyze and process the pre–post data. The ***Table 1*** on the right presents the intervention study design.

**Table 1:** Intervention study design.


	PRE-TEST	INTERVENTION TREATMENT	POST-TEST

Test Group 1	O_1_	X	O_2_

Test Group 2	O_1_	X	O_2_

Control Group	O_1_	_	O_2_


### Sample and investigation

#### Sample

The initial participants were 83 older adults with MCI diagnosed by the Department of Neurology and Memory Disorder Clinic of Shanghai CZ Hospital.

As evaluated by professional medical staff, the criteria included an MMSE score of ≤ 20 points for those with a primary school education or ≤ 24 points for those with a junior high school education or beyond, or whose MoCA-BJ score was between 15 and 23 points. Eligible patients were diagnosed with MCI using combined biomarker testing; were 55 years old or older; had clear consciousness, normal language expression, and communication skills; had basic daily life ability; did not have major cardiovascular or cerebrovascular diseases such as stroke and cerebral hemorrhage; and did not have schizophrenia, mania, or other severe mental disorders. Under the principle of informed consent, they voluntarily participated in this study, and a family member was required to accompany them during the activity. After the recruitment of group members, patients with MCI who did not participate in the intervention activities subjectively were treated as the control group, with the consent of the patients and their family members.

#### Investigation steps

This study involved a comprehensive baseline survey of patients in the CZ Hospital’s Cognitive Impairment Clinic Patient Bank using the simple Mental State Examination Scale (MMSE) and the Montreal Cognitive Test Scale Beijing Version (MoCA-BJ). Period, three participants in the two test groups dropped out and 20 participants in the control group dropped out. This left 19 participants in each test group (38 total) and 16 in the control group (see attachment for basic information on the research participants).

#### Intervention target

This study assessed the ability of group work methods in social work and effective interventions to delay the decline of patients’ cognitive function and improve their quality of life. The detailed intervention categories are listed in ***Table 2***.

**Table 2:** Non-pharmacological Intervention approach summary.


SESSIONS	INTERVENTION APPROACH	SESSION TARGET

No. 1 Sailing together	Group dynamics	Get the members connected and make the group contract; Needs assessment responses and group introduction.

No. 2 Knowing your brain	Cognitive training	Number calculation training and group puzzles; Color and shape riddles competition in pairs.

No. 3 Music Magic	Music Therapy	Music therapy introduction and practice; Meditation and relax exercise;Positive communication with family.

No. 4 Reactivate the functions	Cognitive training	Master the associative memory approach; Further group cohesion building.

No. 5 Hands and Brian	Therapeutic touch	Pain release with family members; Facilitate the positive communication between family members.

No. 6 Life creations	Creative story telling	Facilitate the creative and expressive ability; Facilitate the mutual communication in the group.

No. 7 Recall and recharge	Reminiscence therapy	Enhance the self-esteem and self-efficacy; Enhance the language and communication ability.

No. 8 Moving forward together	“4F”Reflection Model	Promote the teamwork ability; Reflect on previous session gaining;Build up hope and strengthen the group affiliation and group support.


This project and study sought to achieve the following aims:

1. Disseminate knowledge about cognitive disorders to patients with cognitive impairment and their families, establish scientific cognition, and establish a rational attitude.
2. Through group intervention, lead family members of patients to master daily training methods that can effectively delay the cognitive decline of patients with MCI.
3. Relieve patients’ negative psychological emotions caused by illness and help them maintain a positive and optimistic attitude toward life.
4. Through group interaction, help patients establish new social connections, enhance self-esteem and self-efficacy, and recover social functions such as social interaction.
5. Increase benign communication between patients and caregivers and help family members establish a support network, thereby reducing pressure on caregivers.

## Results

### General statistics of samples

The total effective sample size of the test group and control group was 54 people, and the average age was 73.78 years old. Among them, 48.1% were female patients and 51.9% were male patients. Regarding education, 11.1% had completed elementary school or below, 20.4% had completed junior high school (technical secondary school), 33.3% had completed high school (technical school), 33.3% earned a bachelor’s degree (college), and 1.9% had a master’s degree or beyond. All participants were patients with MCI diagnosed both by scale measurement and a doctor. The effective sample size of each test group was 19 people; the effective sample size of the control group was 16 people.

An independent samples *t*-test was used to compare differences between the two test groups and the control group in terms of gender, age, education level, work status, etc.; no statistically significant differences emerged between the groups (*p* > .05). The ***Table 3*** illustrates the statistical comparison between the test group and control group.

**Table 3:** Comparison of general data between Test Group and control group.


TERMS	TEST GROUP 1 (N = 19)		TEST GROUP 2 (N = 19)		CONTROL GROUP (N = 16)		T1; P1	T2; P2

Gender	Male 11 (58.0%)	Female 8 (42.1%)	Male 10 (52.6%)	Female 9 (47.4%)	Male 7 (43.8%)	Female 9 (56.3%)	–0.818; >0.05	–0.511; >0.05

Age	75.95 ± 5.191		71.42 ± 8.591		74.00 ± 7.581		0.898; >0.05	–0.933; >0.05

Education	Elementary school and below 0 (0%)		Elementary school and below 3 (15.8%)		Elementary school and below 3 (18.8%)		1.455; >0.05	0.692; >0.05

	Junior high school/technical secondary school5 (26.3%)		Junior high school/technical secondary school4 (21.1%)		Junior high school/technical secondary school2 (12.5%)			

	High School/Technical School7 (36.8%)		High School/Technical School3 (15.8%)		High School/Technical School8 (50%)			

	Undergraduate/College6 (31.6%)		Undergraduate/College9 (47.4%)		Undergraduate/College3 (18.8%)			

	Master’s degree and above 1 (5.3%)		Master’s degree and above 0 (0%)		Master’s degree and above 0 (0%)			

Working conditions	Retired		Retired		Retired			


*Note*: t1; p1 is the comparison between test group 1 and control group; t2; p2 is comparison between test group 2 and control group.

### Comparison of pretest data between test group and control group

According to the independent samples *t*-test, the initial scores of the test groups and the control group on the MMSE, MoCA-BJ, ADL, PSQI, GDS, and SSRS did not differ significantly (*p* > .05). The baseline statistics of three groups presents in ***Table 4***.

**Table 4:** Comparison of baseline data of three groups of patients.


SCALE	TEST GROUP 1 N = 19	TEST GROUP 2 N = 19	CONTROL GROUP N = 16	T1; P1	T2; P2

MMSE	22.26 ± 3.914	23.42 ± 4.525	22.88 ± 4.573	–0.427; >0.05	0.354; >0.05

MoCA-BJ	18.83 ± 5.193	18.37 ± 6.085	17.56 ± 5.341	0.703; >0.05	0.412; >0.05

ADL	16.63 ± 4.362	15.53 ± 2.270	16.00 ± 3.266	0.477; >0.05	–0.504; >0.05

PSQI	6.44 ± 4.422	5.95 ± 4.275	6.20 ± 4.814	0.152; >0.05	–0.162; >0.05

GDS	9.37 ± 6.157	8.21 ± 6.746	6.19 ± 5.540	1.593; >0.05	0.957; >0.05

SSRS	35.37 ± 7.960	32.74 ± 6.514	36.81 ± 8.199	–0.527; >0.05	–1.639; >0.05


*Note*: t1; p1 is the comparison between test group 1 and control group; t2; p2 is comparison between test group 2 and control group.

### Change trend of the pretest and posttest between the test and control groups

After two months of intervention, changes in data measured at the end of the third month relative to baseline show that for many patients in Test Group 1, their cognitive ability (e.g., MoCA-BJ scale score) improved or remained stable (76.3%), daily living ability (ADL) improved or remained stable (68.4%), sleep quality (PSQI) improved or remained stable (57.9%), depression level (GDS) decreased or remained stable (68.5%), and social support (SSRS) increased or remained stable (26.3%). In Test Group 2, cognitive ability improved or remained stable (89.5%), daily living ability improved or remained stable (78.9%), sleep quality improved or remained stable (84.2%), depression level decreased or remained stable (68.5%), and social support increased or remained stable (73.7%). In the control group, the participants’ cognitive ability improved or remained stable (43.7%), daily living ability improved or remained stable (62.5%), sleep quality improved or remained stable (56.3%), depression level decreased or remained stable (25.1%), and social support increased or remained stable (50%). The changes of the pre-test and post-test within both test and control groups are listed in ***Table 5***.

**Table 5:** The changes of the pre-test and post-test between the test group and control group.


CHANGES OF SCORE		TEST GROUP 1 (%)	TEST GROUP 2 (%)	CONTROL GROUP (%)

MMSE	Unchanged or improved	68.4	68.4	31.2

	Negative development	31.6	31.6	68.8

MoCA-BJ	Unchanged or improved	68.4	89.5	43.7

	Negative development	31.6	10.5	56.3

ADL	Unchanged or improved	68.4	78.9	62.5

	Negative development	31.6	21.1	37.5

PSQI	Unchanged or improved	57.9	84.2	56.3

	Negative development	42.1	15.8	43.8

GDS	Unchanged or improved	68.5	68.5	25.1

	Negative development	31.5	31.5	74.9

SSRS	Unchanged or improved	26.3	73.7	50.0

	Negative development	73.7	26.3	50.0


This indicates that most test group members showed a trend of improving or unchanged cognitive impairment, and the number of people with worsening impairment was far less than that of the control group. Therefore, to a certain extent, this suggests that our intervention is effective in delaying the speed of cognitive decline. In terms of daily living ability, the test groups fared significantly better than the control group. In terms of sleep quality, improvement in the test groups was slightly higher than that of the control group. Regarding depression or potential depression, the test groups improved significantly, whereas the control group declined, indicating the intervention can help patients maintain a good and positive attitude, improve mood, and reduce the risk of depression. In terms of social support and utilization, the test groups’ situation had not improved, suggesting that the current integrated intervention procedures are not effective in improving social support and utilization.

### Comparison of pretest data between test and control groups

#### Test Group 1 and control group

According to our statistical analysis, in terms of cognitive level, Test Group 1 had significant improvement after the intervention, whereas the control group had a decrease on average, indicating that the intervention can significantly improve cognitive level (MMSE; *p* < .05). In terms of abilities of daily living, Test Group 1 had a slight increase in life dependence after the intervention; a parallel increase in dependence in the control group was greater, although the difference between the groups was not significant. In terms of sleep quality, the overall level of the two groups of patients had improved to a certain extent, but improvement in Test Group 1 was higher and the difference between the groups was not significant. In terms of depression, the overall level of depression in the two groups increased slightly, with a greater increase in the control group but no significant difference between the groups. In terms of social support, there was no significant difference between the two groups, which suggests that the intervention has no significant effect on social support. The comparison between test group 1 and control group is presented in ***Table 6***.

**Table 6:** Comparison between test group 1 and control group.


ΔT	TEST GROUP 1 N = 19	CONTROL GROUP N = 16	T	P

MMSE	1.63 ± 3.131	–1.31 ± 3.092	2.787	<0.05

MoCA-BJ	0.22 ± 3.078	–0.69 ± 2.983	0.873	>0.05

ADL	0.89 ± 5.724	1.63 ± 2.895	–0.462	>0.05

PSQI	–1.00 ± 3.500	–0.73 ± 3.845	–0.205	>0.05

GDS	0.37 ± 6.601	0.63 ± 4.978	–0.128	>0.05

SSRS	–3.58 ± 5.891	–3.56 ± 6.985	–0.008	>0.05


*Note*: ΔT is the average score of the pre-test and post-test for each scale.

#### Test Group 2 and control group

Regarding cognitive level, Test Group 2 had significant improvement after the intervention, whereas the control group had a decrease on average, indicating that the intervention can significantly improve cognitive level (MoCA-BJ; *p* < .05). In terms of ability of daily living, life dependence in Test Group 2 increased slightly after the intervention; although the dependence of the control group increased more severely, the difference between the groups was not significant. In terms of sleep quality, the overall level of the two groups had improved to a certain extent, the improvement in Test Group 2 was higher, and the difference between the groups was not significant. The overall level of depression in Test Group 2 decreased, whereas participants in the control group had a slight tendency to develop depression; the difference between the groups was not significant. The level of social support in Test Group 2 increased and the level of social support in the control group decreased. The intervention had a significant effect on improving social support (SSRS; *p* < .05). The above ***Table 7*** illustrates the comparison between test group 2 and the control group.

**Table 7:** Comparison between test group 2 and control group.


ΔT	TEST GROUP 2 N = 19	CONTROL GROUP N = 16	T	P

MMSE	0.37 ± 3.467	–1.31 ± 3.092	1.500	>0.05

MoCA–BJ	1.79 ± 2.955	–0.69 ± 2.983	2.460	<0.05

ADL	0.26 ± 3.142	1.63 ± 2.895	–1.324	>0.05

PSQI	–0.95 ± 2.505	–0.73 ± 3.845	–0.196	>0.05

GDS	–2.21 ± 6.015	0.63 ± 4.978	–1.501	>0.05

SSRS	1.68 ± 4.083	–3.56 ± 6.985	2.648	<0.05


*Note*: ΔT is the average score of the pre-test and post-test for each scale.

### Comparison of pretest and posttest between test groups

#### Test Group 1

In terms of cognitive level, Test Group 1 had a significant increase in scores before and after the intervention, indicating that the intervention can significantly improve cognitive level (MMSE; *p* < .05). Other changes included a slight increase in life dependence, improved overall sleep quality, and slightly higher depression, although the differences were not significant. The level of social support increased from baseline, indicating the intervention can significantly increase the level of social support (SSRS; *p* < .05). Changes between pre-test and post-test of test group 1 is shown in ***Table 8***.

**Table 8:** Comparison of pre-test and post-test of test group 1.


TERMS	MEAN		T	P

	BEFORE INTERVENTION	AFTER INTERVENTION		

MMSE	22.26 ± 3.914	23.89 ± 3.957	–2.272	<0.05

MoCA–BJ	18.83 ± 5.193	19.06 ± 5.493	–0.306	>0.05

ADL	16.63 ± 4.362	17.53 ± 5.853	–0.681	>0.05

PSQI	6.71 ± 4.413	5.71 ± 3.549	1.178	>0.05

GDS	9.37 ± 6.157	9.74 ± 6.838	–0.243	>0.05

SSRS	35.37 ± 7.960	31.79 ± 5.523	2.648	<0.05


#### Test Group 2

Regarding cognitive level, Test Group 2 had a significant increase in scores before and after the intervention, indicating the intervention can significantly improve cognitive level (MoCA-BJ; *p* < .05). Other changes included a slight increase in life dependence, improvements to overall sleep quality, a slight increase in depression, and an increase in social support, but the differences were not statistically significant. Changes of pre-test and post-test of test group 2 is shown in ***Table 9***.

**Table 9:** Comparison of pre-test and post-test of test group 2.


TERMS	MEAN			

	BEFORE INTERVENTION	AFTER INTERVENTION	T	P

MMSE	23.42 ± 4.525	23.79 ± 4.341	–0.463	>0.05

MoCA–BJ	18.37 ± 6.085	20.16 ± 5.480	–2.640	<0.05

ADL	15.53 ± 2.270	15.79 ± 3.765	–0.365	>0.05

PSQI	5.95 ± 4.275	5.00 ± 4.055	1.649	>0.05

GDS	8.21 ± 6.746	6.00 ± 4.830	1.602	>0.05

SSRS	32.74 ± 6.514	34.42 ± 6.310	–1.798	>0.05


### Comparison of pretest and posttest of control group

In the control group, the scores of the posttest were lower than those of the pretest for cognitive ability, but the difference was not significant. In terms of daily life ability, the comparison found that life dependence had increased significantly (l ADL; *p* < .05). Other changes included improved sleep ability, increased depression, and decreased social support, but the differences were not significant. The comparison of pre-test and post-test of control group is shown in ***Table 10***.

**Table 10:** Comparison of pre-test and post-test of control group.


TERMS	MEAN		T	P

	BEFORE INTERVENTION	AFTER INTERVENTION		

MMSE	22.88 ± 4.573	21.56 ± 5.202	1.698	>0.05

MoCA–BJ	17.56 ± 5.341	16.88 ± 6.087	0.922	>0.05

ADL	16.00 ± 3.266	17.63 ± 5.414	–2.245	<0.05

PSQI	6.20 ± 4.814	5.47 ± 3.292	0.739	>0.05

GDS	6.19 ± 5.540	6.81 ± 3.060	–0.502	>0.05

SSRS	36.81 ± 8.199	33.25 ± 7.353	2.040	>0.05


## Conclusion and Discussion

First, findings highlight the need to promote the hospital–family dual care model. Patients with MCI, because of their mild illness, often do not need hospitalization, but only need outpatient clinic care to receive medication and regular follow-up care. Medical personnel can only provide limited services, and the patient’s abilities and the role of social support sources such as relatives and friends have not been taken seriously. Through the establishment of a hospital–family dual care model, medical social workers can help patients form a sense of hope for the future and master effective training methods in about two months. After returning to their family, patients can still carry out consolidation training to delay disease development.

The secoend major finding is that during related research involving older adults with MCI, the concept of holistic care should be promoted and developed. Taking the biopsychosocial medicine model as their theoretical guide, researchers should pay attention to not only the effect of drug intervention, but also the needs of patients at all levels, actively respond to them, and strive to improve the quality of life and self-efficacy of older patients.

This study verifies the effectiveness of social work group interventions in improving the cognitive level of patients with MCI and delaying the development of the disease. Therefore, for social workers, the practical design of this study can be used for reference.

Because the group activities were relatively limited—lasting for eight sessions across two months—the intervention effect requires us to think more about the intensity and continuity of training. Therefore, by enhancing the subjective initiative of patients, they can practice autonomously at home. On the other hand, in addition to online WeChat group training supervision, strategies might involve designing applications or mini programs to help patients check in on basic intervention content and form good habits. For patients who struggle to use smartphones, approaches might even involve designing smart physical products in combination with intervention content. In addition, follow-up interventions can focus on the therapeutic factors proposed in this study to enhance the group effect.

Due to factors such as resources and capabilities, the sample size of this study was relatively small; a long-term research mechanism is expected to be established. In the future, researchers should compile the service activity design and implementation process into a standardized operation manual to ensure trained social workers (especially medical social workers) can strictly implement interventions for patients with MCI with no significant differences in baseline values and contribute to a shared database, thus providing better empirical evidence.

The MoCA and MMSE scales used in this study have high medical reliability and validity but low sensitivity for patients with MCI. Future research should test the Self-Rating Depression Scale, General Self-Efficacy Scale, and other instruments.

In terms of research on intervention effects, continuous observation and measurement should be carried out with intervention participants for longer (such as 6 months or 1 year after intervention). In addition, more attention needs to be paid to the recording and analysis of key events to reduce the impact of irrelevant variables on the research results.

Overall, the development of this research provides a way of thinking and solutions for older adults to achieve better health in later life. It has positive practical significance and provides relevant guidance for evidence-based social work interventions for people with MCI, particularly social workers and medical social workers. In the future, social workers should devote themselves to exploring more diversified, systematic, and highly operable nootropic intervention models for early stage patients and to studying and recording the comprehensive clinical effects of such interventions on older patients with MCI to reduce the occurrence of dementia, slow the development of the disease, and improve the quality of life of older adults with MCI.
